# Deep Learning-Assisted Microscopic Polarization Inspection of Micro-Nano Damage Precursors: Automatic, Non-Destructive Metrology for Additive Manufacturing Devices

**DOI:** 10.3390/nano15110821

**Published:** 2025-05-29

**Authors:** Dingkang Li, Xing Peng, Zhenfeng Ye, Hongbing Cao, Bo Wang, Xinjie Zhao, Feng Shi

**Affiliations:** 1 College of Intelligent Science and Technology, National University of Defense Technology, Changsha 410073, China; lidingkang22@nudt.edu.cn (D.L.); yezhenfeng22@nudt.edu.cn (Z.Y.); wangbonudt@nudt.edu.cn (B.W.); zhaoxinjie24@nudt.edu.cn (X.Z.); shifeng@nudt.edu.cn (F.S.); 2 National Key Laboratory of Equipment State Sensing and Smart Support, Changsha 410073, China; 3 Hunan Provincial Key Laboratory of Ultra-Precision Machining Technology, Changsha 410073, China

**Keywords:** additive manufacturing, deep learning, micro-nano damage precursor, defect detection, non-destructive inspection

## Abstract

Additive Manufacturing (AM), as a revolutionary breakthrough in advanced manufacturing paradigms, leverages its unique layer-by-layer construction advantage to exhibit significant technological superiority in the fabrication of complex structural components for aerospace, biomedical, and other fields. However, when addressing industrial-grade precision manufacturing requirements, key challenges such as the multi-scale characteristics of surface damage precursors, interference from background noise, and the scarcity of high-quality training samples severely constrain the intelligent transformation of AM quality monitoring systems. This study proposes an innovative microscopic polarization YOLOv11-LSF intelligent inspection framework, which establishes an automated non-destructive testing methodology for AM device micro-nano damage precursors through triple technological innovations, effectively breaking through existing technical bottlenecks. Firstly, a multi-scale perception module is constructed based on the Large Separable Kernel Attention mechanism, significantly enhancing the network’s feature detection capability in complex industrial scenarios. Secondly, the cross-level local network VoV-GSCSP module is designed utilizing GSConv and a one-time aggregation method, resulting in a Slim-neck architecture that significantly reduces model complexity without compromising accuracy. Thirdly, an innovative simulation strategy incorporating physical features for damage precursors is proposed, constructing a virtual and real integrated training sample library and breaking away from traditional deep learning reliance on large-scale labeled data. Experimental results demonstrate that compared to the baseline model, the accuracy (P) of the YOLOv11-LSF model is increased by 1.6%, recall (R) by 1.6%, mAP50 by 1.5%, and mAP50-95 by 2.8%. The model hits an impressive detection accuracy of 99% for porosity-related micro-nano damage precursors and remains at 94% for cracks. Its unique small sample adaptation capability and robustness under complex conditions provide a reliable technical solution for industrial-grade AM quality monitoring. This research advances smart manufacturing quality innovation and enables cross-scale micro-nano damage inspection in advanced manufacturing.

## 1. Introduction

Additive manufacturing (AM), as a novel and fast-developing method for producing parts, enables the fabrication of complex geometries and customized components with minimal material waste compared to traditional “subtractive” and “equal-material” manufacturing methods. Its layer-by-layer deposition process offers huge flexibility, allowing parts to be precisely built according to pre-designed digital models [1,2]. As a “bottom-up” material accumulation method for fabricating parts, AM imposes new requirements in terms of process technology, materials, production environments, process monitoring, and industrial chains. It represents a disruptive innovation in traditional manufacturing and has garnered extensive attention from both industry and academia. Governments worldwide have formulated policy frameworks to prioritize its development, such as “Made in China 2025” (China), the “National Strategic Plan for Advanced Manufacturing” (USA), and the “Industrial 4.0 Strategic Plan Implementation Recommendations” (Germany). Under these national strategies, AM has been elevated to a critical developmental focus, giving rise to diverse manufacturing technologies. Currently, AM is widely applied across aerospace, transportation, nuclear power, defense, medical devices, energy, automotive manufacturing, and other fields [3].

As the most cutting-edge and challenging technology within the AM system, metal AM technology represents a critical direction for advanced manufacturing development. It is poised to become a key pathway for achieving generational leaps in the structural performance of high-end industrial equipment. Current research on metal AM primarily focuses on four fabrication methods: Selective Laser Melting (SLM), Laser Metal Deposition (LMD), Electron Beam Melting (EBM), and Wire Arc Additive Manufacturing (WAAM) [4]. However, the unique fabrication process of metal additive manufacturing components inherently induces damage precursors—such as low-density zones, residual stresses, and cracks—that degrade device performance. Pore-type damage precursors typically range from 5 µm to 20 µm in size, elongated damage precursors measure approximately 50 µm to 500 µm, and crack-type precursors exhibit lengths and openings generally below 100 µm. The presence of these precursors poses significant safety risks to precision-critical industries. Consequently, detecting and identifying various damage precursor types in metal AM components is crucial, as it not only enhances material utilization but also ensures operational safety in industrial applications. Traditional non-destructive evaluation (NDE) methods for damage precursors include X-ray inspection, ultrasonic testing, liquid penetrant testing, magnetic particle testing, and eddy current testing [5]. Although traditional methods provide high detection accuracy, they suffer from elevated costs, operational complexity, and an inability to reconcile inspection precision with speed. In certain cases, these limitations may fail to fully meet practical production requirements. Consequently, it is imperative to develop a more efficient and cost-effective damage precursor inspection method.

In recent years, deep learning technologies have grown rapidly, with diverse machine learning and deep learning techniques being applied to the monitoring of AM processes. This trend has driven a gradual convergence toward smart manufacturing, paving the way for a hopeful future in intelligent industrial production [6,7,8]. To detect damage precursors on liquid crystal display (LCD) panels, Chen et al. proposed a lightweight YOLO-ADPAM inspection method based on YOLOv4, incorporating an attention mechanism. First, they designed a K-means-CIoU++ clustering algorithm to cluster anchor box sizes within the damage dataset, enabling more accurate and stable bounding box regression. Subsequently, a parallel attention module was introduced, integrating the strengths of channel and spatial attention mechanisms to effectively enhance the network’s detection accuracy [9]. To address the issue of slow detection speed in wind turbine surface damage precursor models, Zhang proposed a lightweight YOLOv5s-based inspection model. The YOLOv5s backbone network was replaced with a MobileNetv3 lightweight network for feature extraction, harmonizing and balancing the model’s lightweight design and accuracy. The final implementation achieved a 5.51% improvement in mean average precision and a 10.79 frames-per-second reduction in detection time compared to the original YOLOv5s model [10].

Although existing deep learning-based methods have achieved high detection accuracy, they still face challenges in acquiring large-scale training samples and high annotation costs in practical applications. To address this, Zhu et al. utilized transfer learning by pre-training on an open-source damage precursor dataset (NEU-DEF). After transferring model parameters, they fine-tuned the model using their dataset of 240 small-sample objects, achieving a final mAP50 of 62% [11]. In the inspection of damage precursors in photovoltaic (PV) cells, Wang et al. adopted the YOLOX model as the backbone network and implemented a transfer learning strategy to accelerate model convergence, mitigating accuracy limitations caused by the limited sample size of damage precursors. This method achieved promising results on the Photovoltaic Electroluminescence Anomaly Inspection dataset (PVEL-AD), attaining a mAP of 96.7% and a detection speed of 71.47 FPS [12]. Li et al. integrated real experimental images obtained from a shearing imaging device to execute and discuss hybrid training strategies for deep learning-based damage precursor inspection. Their work demonstrated that, even with a limited number of experimental training images, the generalization capability of the deep learning network can be significantly enhanced without using any real damage precursor samples, shearing systems, or artificially generated simulated datasets [13]. To further improve the detection accuracy and speed of surface damage precursors in AM components, this study proposes the following enhancements to the YOLOv11 network architecture: (1) The Large Separable Kernel Attention is introduced into the original C2PSA module in the backbone, enhancing the model’s multi-scale adaptability and focus on target regions while suppressing interference from complex backgrounds on the component surfaces. (2) A Slim-neck structure is designed to reduce the model’s complexity without sacrificing accuracy, thereby accelerating detection speed. (3) For training, a hybrid strategy combining real damage precursor images captured by a custom hardware platform with simulated data is adopted, which addresses the challenge of acquiring sufficient training samples.

The main contributions of this paper are as follows:

1. The LSKA technique was incorporated into the Backbone by integrating it into the C2PSA module of YOLOv11. This configuration enhances the model’s performance in three dimensions: improving multi-scale target detection capability, strengthening focus on critical regions, suppressing interference from complex background information, and ultimately achieving synergistic optimization of detection accuracy and classification performance.
2. The construction of Slim-neck through the introduction of GSConv and VoV-GSCSP modules, which reduces the complexity of convolutional operations while effectively fusing feature maps from different stages. This design accelerates detection speed without losing accuracy, making the network more adaptable to real-time inspection requirements.
3. Synthetic damage precursor images are generated based on precursor features extracted from AM component surfaces using a hardware system. These synthetic images are combined with real captured damage precursor images to form a hybrid training dataset, effectively solving the challenge of not having enough training samples for damage precursor inspection networks.
4. This study innovatively integrates polarization imaging technology with deep learning algorithms, breaking the limitations of traditional detection methods imposed by target surface temperature and high-reflectance phenomena. The proposed approach effectively suppresses background clutter interference and achieves clear extraction of damage precursor features in complex environments. Through dual optimization of high-precision detection and geometric texture information reconstruction, the accuracy and reliability of inspection results are significantly enhanced. This technological breakthrough establishes a crucial foundation for quality control and safety assurance in related fields.

The structure of this paper is organized as follows: Section 2 covers the surface damage precursor acquisition system of AM components, the hybrid training strategy integrating collected and synthetically generated precursor features, and the implementation methodology for damage precursor inspection experiments. Section 3 breaks down the YOLOv11 model, the integration of the LSKA attention mechanism, and the proposed YOLOv11-LSF model with the Slim-neck architecture. Section 4 presents the experiments on damage precursor inspection model training and analyzes the results. Section 5 summarizes the key findings and discusses future research directions.

## 2. Methodology

### 2.1. Microscopic Polarization Damage Precursor Inspection System

The inspection system includes illuminating equipment, optical components, sensors, and signal processing units commonly. The selection of imaging methods determines the efficiency and accuracy of damage precursor inspection. During the inspection of damage precursors, the complex working conditions, uneven light distribution, and surface reflection of metal components can cause high-reflectance surfaces to produce bright areas. These bright areas obscure the inherent geometric texture information of the inspection targets, making it impossible to accurately restore the corresponding contour morphology information. In contrast, polarization imaging is not affected by the surface temperature or high reflectance of the inspection targets. It can avoid interference from background clutter, effectively highlight damage precursors against the background, and solve the problem of high-reflectance areas obscuring damage precursors and making it difficult to accurately restore geometric texture information. Thus, to solve this problem effectively, we propose a microscopic polarization damage precursor inspection system (MPIS) based on polarized machine vision and deep learning.

**Figure 1:**
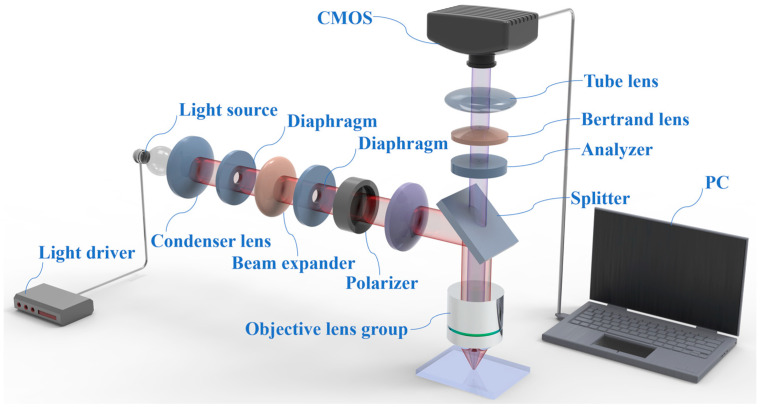
The microscopic polarization damage precursor inspection system.

Figure 1 illustrates the schematic diagram of the proposed damage precursor inspection system setup named microscopic polarization damage precursor inspection system (MPIS) for short. The system consists of two main parts: the microscopic polarization imaging subsystem, and the automatic deep learning processing subsystem. The microscopic polarization imaging subsystem is based on the objective lens group, the light source, the Bertrand lens, the tube lens, the condenser lens, the diaphragm, the beam expander, the polarizer, the analyzer, and the high-resolution CMOS. The light source is a 12 V 30 W halogen lamp, and its brightness can be adjusted to adapt to different materials. Both the polarizer and analyzer can achieve 360° free rotation. The objective lens group is designed with a dual telecentric achromatic structure, with a working distance of approximately 20 mm, a numerical aperture of 0.25, and a magnification of 10 times. The pixel size of CMOS is 2.4 microns, and the chip size is 1 inch. The automatic deep learning processing subsystem mainly focuses on damage precursor data augmentation, identification, and classification.

### 2.2. Damage Precursor Data Classification and Augmentation

**Figure 2:**
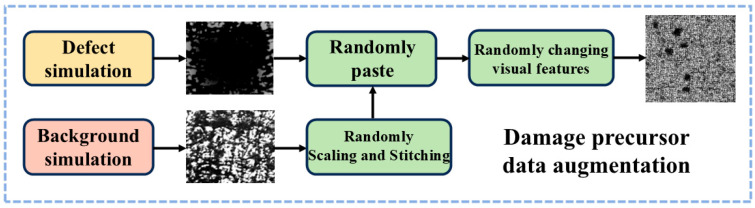
Simulation workflow of porosity-related damage precursors.

In this study, the training images were acquired by the acquisition system described in Section 2.1. We primarily collected two common types of damage precursor images: porosity and crack. The distribution of each category and precursor features is summarized in Table 1. In this study, the simulation of both the background and damage precursors in the images is based on the acquired precursor features. Taking porosity as an example, its simulation process is illustrated in Figure 2. For the background simulation of porosity precursors, we cropped regions without precursors from the collected data. For the porosity precursor simulation, 100 Gaussian functions with distinct variances and means were randomly generated within a 100×100 grid. These functions were superimposed to create a randomly fluctuating 100×100 matrix. Subsequently, the matrix was scaled to the range (0, 255), and its 100×100 dimensions were randomly resized to simulate precursors of varying sizes. Finally, multiple coordinates were randomly generated within a 3000×3000 grid to assign positions for the simulated precursors in the background.

**Figure 3:**
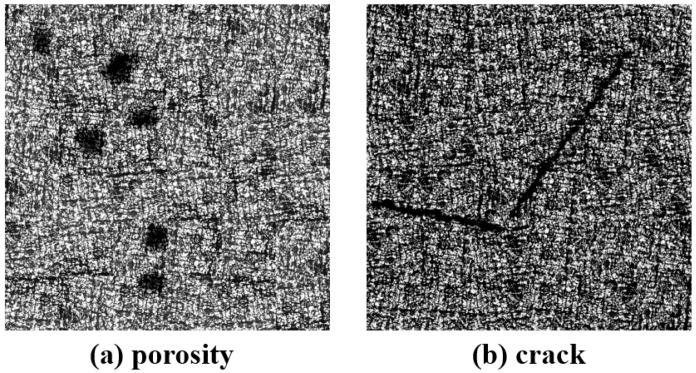
Porosity and crack damage precursor simulation images.

The simulation process for crack damage precursors follows a methodology similar to that of porosity, both based on the acquired image features for synthetic reconstruction. The final simulated images of porosity and crack damage precursors are illustrated in Figure 3.

### 2.3. Inspection Process

**Figure 4:**
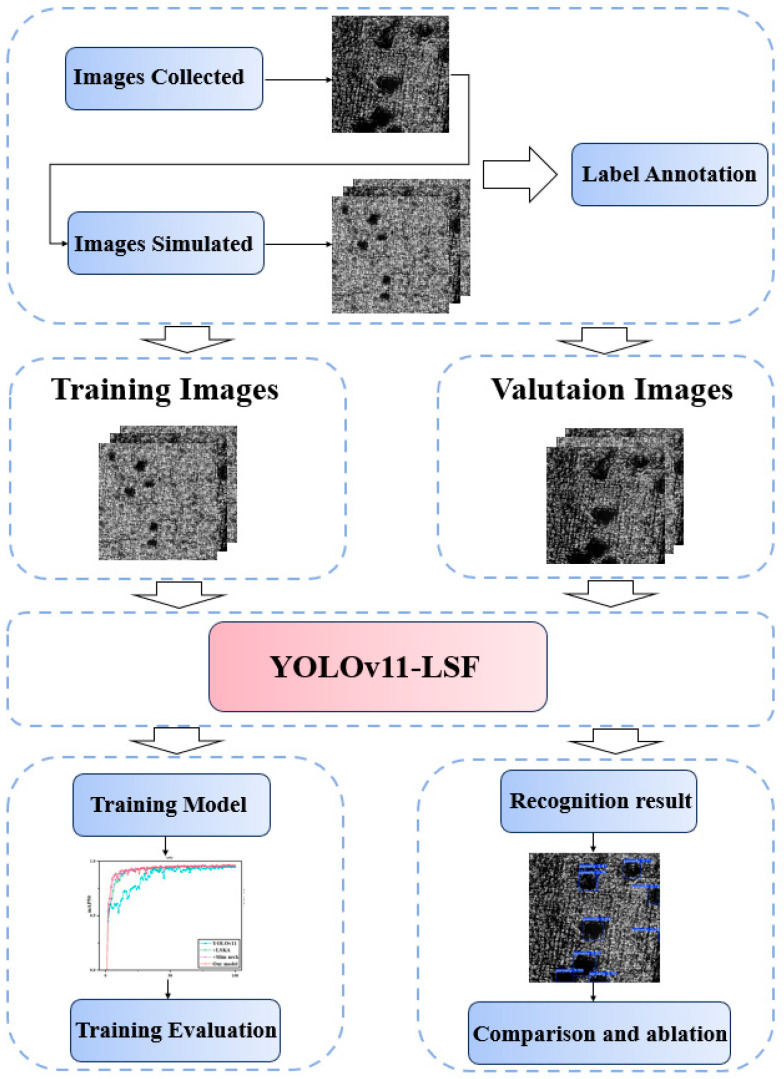
The deep learning-assisted microscopic polarization damage precursor inspection processes.

Figure 4 illustrates the experimental workflow of the proposed YOLOv11-LSF model, which comprises four stages: dataset acquisition, batch image processing, model training, and performance evaluation. First, damage precursor images on the surface of AM components are captured using the hardware acquisition system; synthetic data resembling the captured precursors are then generated through simulation based on their morphological features. Both raw and synthetic images are annotated using LabelImg, saved as TXT files, and combined to form the hybrid training dataset. In the model design phase, the YOLOv11-LSF model is developed to enhance inspection accuracy and efficiency by integrating the LSKA attention mechanism into the backbone, constructing a Slim-neck structure, and training with the hybrid dataset (technical details are discussed in Section 3). During evaluation, a unified test set is adopted to ensure fairness, with performance metrics including precision, recall, mean average precision, GFLOPs, and other critical parameters. Comparative analyses of different models are conducted to validate the effectiveness of the proposed damage precursor inspection framework.

## 3. Proposed Method

### 3.1. YOLOv11 Model

In 2015, Redmon et al. dropped the You Only Look Once (YOLO) algorithm, marking a pivotal breakthrough in the field of object detection [14]. This innovative method, as its name implies, processes the entire image in a single channel to detect objects and their locations. Diverging from the traditional two-stage detection process, the YOLO method frames object detection as a regression problem, thereby simplifying the detection pipeline compared to conventional methods. As the latest iteration in the YOLO series, YOLOv11 builds on the foundation of YOLOv1 and represents a significant leap in real-time object detection technology. Unveiled at the YOLO Vision 2024 (YV24) conference, YOLOv11 shows off the cutting-edge advancements in the field [15]. The release of YOLOv11 has provided a robust foundation for our research. Furthermore, the advanced nature of YOLOv11 signifies its enhanced adaptability and scalability, which are critical to addressing challenges in specific vision tasks. However, we also recognize certain limitations of the YOLOv11 model in practical applications [16]. For example, the model requires further improvement in accuracy and speed for practical applications and is less effective for samples with data acquisition challenges.

**Figure 5:**
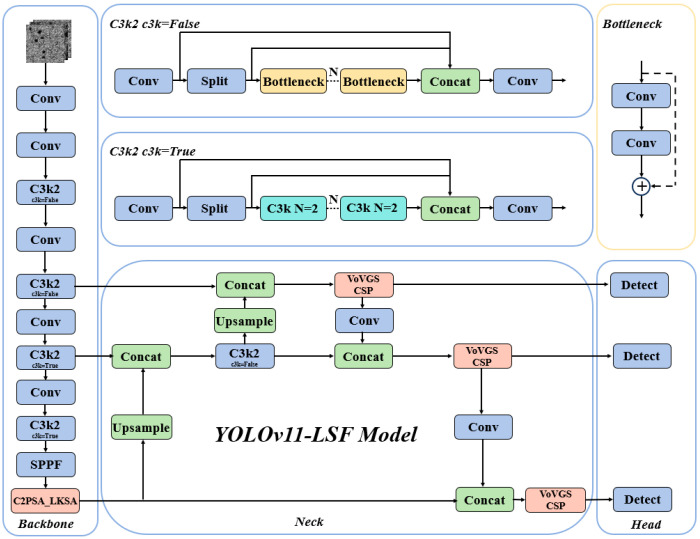
The YOLOv11-LSF network architecture.

These challenges are particularly critical for our research because our objective is to develop an efficient and practical damage inspection system. Furthermore, while processing targets with significant scale variations, such as pores and scratches, the detection accuracy of YOLOv11 still needs improvement. This indicates that while YOLOv11 performs well in many aspects, more optimizations and adjustments are required for specific applications, such as AM damage precursor detection. This study proposes two improvements to the YOLOv11 network architecture. The enhanced architecture primarily comprises three components: the Backbone network, Neck network, and Head network, with its model structure illustrated in Figure 5. First, the LSKA attention mechanism is integrated into the backbone, which replaces the original C2PSA module with the C2PSA_LKSA module, to enhance multi-scale target detection capabilities and improve the model’s overall detection and classification performance. Second, considering that introducing attention mechanisms may increase model complexity, a lightweight grouped convolution based on the Slim-neck concept is adopted to construct the VoV-GSCSP module, which enables the efficient fusion of feature maps across different stages. This achieves reduced computational complexity without sacrificing accuracy.

### 3.2. C2PSA_LKSA Module

Enhancing AM damage precursor detection requires models to adapt to multi-scale features, especially when identifying targets with extreme size variations like pores and scratches. Large kernel convolutions expand the receptive field and enhance global modeling capabilities, providing a stronger contextual understanding for target detection, which is especially suitable for multi-scale target localization and classification in complex scenarios. However, introducing large-kernel convolutions may lead to quadratic growth in computational and memory overhead, hindering the practical deployment of the model. The principle of LSKA attention addresses the limitations of the traditional Large Kernel Attention (LKA) module in Visual Attention Networks (VAN). The LKA module struggles with high computational and memory requirements when processing large-kernel convolutions. LSKA attention tackles these challenges through innovative kernel decomposition and cascaded convolution strategies, reducing computational and memory costs while preserving efficient image processing capabilities [17]. The main formula of LSKA operation is as follows:

The input feature map $F_{C}\in R^{C\times H\times W}$ Undergoes factorized convolution processing:

(1) $$\begin{array}{c} Z_{C}={DW_{Conv}}_{1\times k}\circ {DW_{Conv}}_{k\times 1}+{DW_{D_{Conv}}}_{1\times k}^{\left(d\right)}\circ {DW_{D_{Conv}}}_{k\times 1}^{\left(d\right)} \end{array}$$

here, ∘ denotes operation cascade, and ${DW\_D\_Conv}^{\left(d\right)}$ represents a depth wise separable convolution with a dilation rate of *d.*

Channel dimension is compressed via 1×1 convolution to generate attention weights:

(2) $$\begin{array}{c} A_{C}=W_{1\times 1}*Z_{C} \end{array}$$

In the formula, $W_{1\times 1}$ denotes the learnable 1×1 convolution kernel, ∗ represents the convolution operation, and the output $A_{C}\in R^{1\times H\times W}$ is the spatial attention map.

Finally, feature enhancement is achieved via Hadamard product:

(3) $$\begin{array}{c} F_{C}=A_{C}⊗F_{C} \end{array}$$

where ⊗ denotes the element-wise product, preserving the original feature resolution.

**Figure 6:**
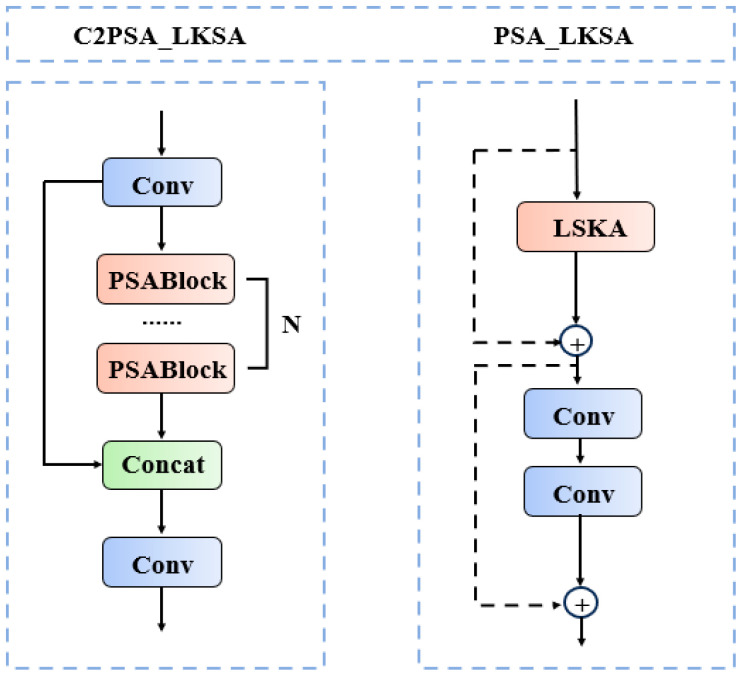
The C2PSA_LKSA module network architecture.

In this study, we introduce the LSKA attention mechanism into the backbone and propose the C2PSA_LKSA module, which enhances the model’s capability to detect multi-scale targets and focus on key regions without introducing excessive parameters. The structure of the C2PSA_LKSA module is illustrated in Figure 6.

The C2PSA is a novel spatial attention module introduced in YOLOv11 after the feature pyramid module to enhance spatial attention in feature maps. This spatial attention mechanism allows the model to focus more effectively on critical regions within images. By spatially aggregating features, the C2PSA module enables YOLOv11 to concentrate on specific regions of interest, thereby potentially improving the detection accuracy for objects of varying sizes and locations. However, the traditional C2PSA module struggles to adapt to targets of varying scales. The introduction of the LKSA attention mechanism effectively addresses this limitation. The LSKA attention mechanism, the core of the C2PSA_LKSA module, differentiates itself from conventional large-kernel convolutions through an innovative strategy of kernel decomposition and cascaded convolutional operations. This approach enhances the model’s ability to detect multi-scale targets and focus on critical regions while avoiding excessive parameter expansion. Additionally, it suppresses interference from complex background information, thereby improving the model’s overall detection and classification accuracy in a computationally efficient manner.

### 3.3. Slim-Neck

While attention mechanisms can effectively enhance a model’s ability to detect multi-scale targets and focus on critical regions, they may inadvertently increase model complexity. To mitigate this issue and reduce complexity without sacrificing accuracy, we introduce *GSConv* (Gather-and-Scatter Convolution) into the neck. Building upon the GSbottleneck, we design a cross-level fractional network module—termed the VoV-GSCSP module—using a single-aggregation strategy. Finally, the Slim-Neck, composed of *GSConv* and VoV-GSCSP, simplifies computational and architectural complexity while maintaining high accuracy. The VoV-GSCSP module proposed in this work constructs a Path Aggregation Feature Pyramid Network, which seamlessly integrates the PAFPN with multi-scale information to achieve comprehensive feature fusion [18]. In the backbone network of CNNs, input images almost always undergo a similar transformation process: spatial information is progressively transferred to the channel dimension. Each spatial compression and channel expansion of feature maps leads to a partial loss of semantic information. Channel-dense convolutions preserve the implicit connections between channels to the greatest extent, whereas channel-sparse convolutions completely sever these connections. *GSConv* optimally preserves these connections while achieving lower computational complexity [19]; its computational process can be decomposed as follows:

(4) $$\begin{array}{c} X_{DSC}=DWConv\left(X\right)⊗W_{pw} \end{array}$$

(5) $$\begin{array}{c} X_{SC}=Conv\left(X\right) \end{array}$$

(6) $$\begin{array}{c} GSConv=\sigma \left(\lambda_{1}\cdot X_{DSC}+\lambda_{2}\cdot X_{SC}\right) \end{array}$$

where DSC denotes Deep Separable Convolution, SC represents Standard Convolution, DWconv stands for Deep Convolution, $W_{pw}$ is the pointwise convolution kernel, $\lambda_{1}$ and $\lambda_{2}$ are learnable channel attention coefficients, and σ denotes the Swish/Mish nonlinear activation function. This dual-branch structure achieves an approximately 60% reduction in theoretical computational cost (FLOPs) compared to standard convolution, while the SC branch compensates for the feature degradation inherent to DSC.

**Figure 7:**
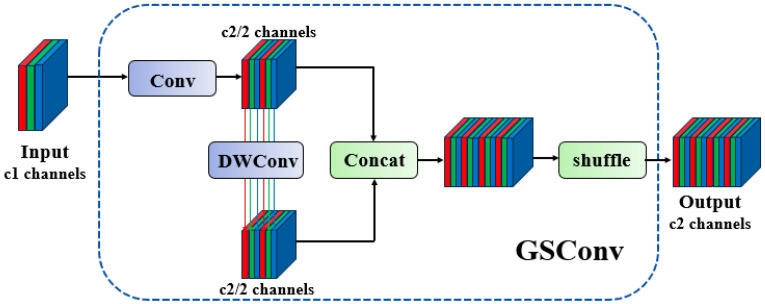
The GSConv architecture.

**Figure 8:**
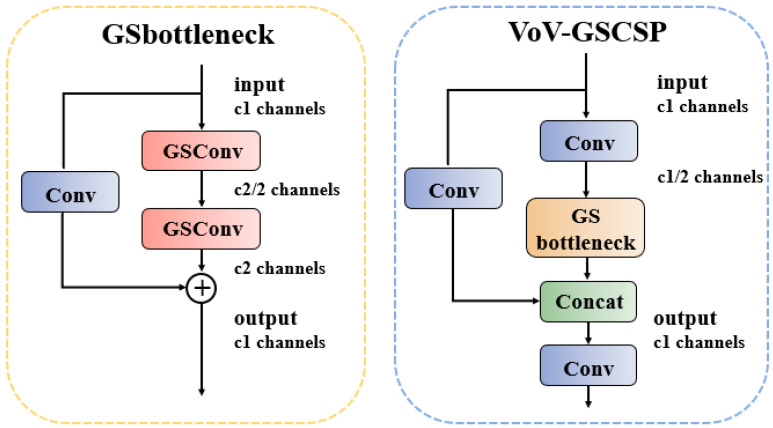
The Slim-neck architecture.

Figure 7 illustrates the data processing pipeline of *GSConv* within a convolutional neural network. The input feature map first passes through a Standard Convolution (SC) layer, followed by a Depthwise Separable Convolution (DSC) layer. Finally, the outputs of these two convolutional layers are concatenated, and a Shuffle Operation is applied to the merged feature map to optimize cross-channel feature representations. In this study, building upon *GSConv*, we further investigate GSbottleneck and VOV-GSCSP, whose architectures are illustrated in Figure 8. The GSbottleneck module is designed to enhance the network’s feature processing capability by stacking *GSConv* modules to amplify model learning capacity. The VoV-GSCSP module adopts divergent structural designs to improve feature utilization efficiency and network performance, with its computational workflow defined as follows:

(7) $$\begin{array}{c} X_{split}=Split\left(X_{in}\right) \end{array}$$

(8) $$\begin{array}{c} X_{bottleneck}={Stack\left[{GSConv}_{3\times 3}\to {GSConv}_{1\times 1}\right]}^{n}\left(X_{split}^{\left(1\right)}\right) \end{array}$$

(9) $$\begin{array}{c} X_{out}=Concat\left(X_{bottleneck},X_{split}^{\left(2\right)}\right)⊗W_{fusion} \end{array}$$

The Split operation divides the input channels into two parts, processes only the primary branch through n GSbottleneck operations, and finally achieves feature fusion via a 1×1 convolution.

These module designs embody the Slim-neck philosophy, aiming to reduce computational complexity and inference time while maintaining accuracy. Through such modular design, the network architectures can be flexibly constructed to suit specific tasks as needed.

## 4. Experiments and Discussion

### 4.1. Dataset

In this study, the model was trained using a mixed dataset composed of real-world captured data and simulated data. Specifically, 64 images containing pores and scratches collected from hardware devices are mixed with 2000 simulated images to form the training dataset. Each damage precursor instance in the images was precisely annotated with bounding boxes, providing detailed information about the target location, size, and category. The dataset was randomly divided into a training set and a validation set at an 8:2 ratio.

### 4.2. Experiment Setting

The experiments in this study were conducted on the PyTorch deep learning framework within an Anaconda virtual environment. Table 2 summarizes the experimental hardware and software configurations, while Table 3 details the key hyperparameter settings used in the experiments.

### 4.3. Performance Metrics

In this study, the model was evaluated using accuracy, recall, mean Average Precision, and GFLOPs to validate the practical effectiveness of the proposed YOLO v11-LSF model in real-world applications [20].

#### 4.3.1. Precision

Accuracy refers to the probability that all detected targets are correctly identified, and its mathematical formulation is provided in Equation (10).

(10) $$\begin{array}{c} Precison=\frac{TP}{TP+FP} \end{array}$$

In the equation, *TP* (True Positives) denotes the number of positive instances correctly predicted, while *FP* (False Positives) represents the number of negative instances incorrectly predicted as positive.

#### 4.3.2. Recall

*Recall* refers to the probability of correctly identifying all positive samples and its mathematical formulation is provided in Equation (11).

(11) $$\begin{array}{c} Recall=\frac{TP}{TP+FN} \end{array}$$

*TP* denotes the number of positive samples correctly predicted, while *FN* represents the number of negative samples incorrectly predicted.

#### 4.3.3. AP

Average Precision (*AP*), which comprehensively integrates variations in Precision and Recall, characterizes the area under the Precision–Recall curve. It is a critical metric for evaluating the performance of an object detection model, and its mathematical formulation is provided in Equation (12).

(12) $$\begin{array}{c} AP=\frac{\sum P_{ri}}{\sum r} \end{array}$$

where $P_{ri}$ is the *P* value corresponded by *ri* on the PR curve, and ∑r=1.

#### 4.3.4. mAP

The *mAP* denotes the mean of Average Precision values, serving as a comprehensive metric that integrates Precision, Recall, and Average Precision. mAP50 represents the *mAP* value under a 50% IoU threshold. A higher *mAP* value reflects greater model accuracy, and its mathematical formulation is provided in Equation (13).

(13) $$\begin{array}{c} mAP=\frac{AP}{num_{classes}} \end{array}$$

The *mAP* metric ranges within 0–1, where a value closer to 1 indicates better performance. This metric is widely regarded as the most critical objective detection indicator in algorithms

#### 4.3.5. GFLOPs

The GFLOPs (Giga Floating Point Operations) is a critical metric for evaluating the computational complexity of neural network models, representing billions of floating-point operations executed per second. A higher GFLOPs value indicates that the model requires more computational resources during execution, which may increase demands on hardware resources and potentially impact training speed and inference efficiency.

### 4.4. Experiment Evaluation

This section analyzes the training results of the model on the hybrid dataset from four perspectives: quantitative experiments, ablation studies, model loss comparisons, and detection performance for different damage precursors. First, in the quantitative experiments, YOLOv11-LSF is compared with recent mainstream YOLO-series models, and the rationale for selecting YOLOv11 as the baseline in this study is elaborated. Second, in the ablation studies, the effectiveness of the proposed improvement modules is validated through experimental data analysis of individual design modifications. Subsequently, the training loss curves of the YOLOv11 baseline and our improved model are compared, demonstrating the improvements in stability and convergence achieved by the refined architecture. Finally, to highlight the practical performance of the enhanced model, tests are conducted on damage precursor images using both the baseline and YOLOv11-LSF, with quantitative results underscoring the superiority of the proposed approach [21].

#### 4.4.1. Quantitative Analysis

**Figure 9:**
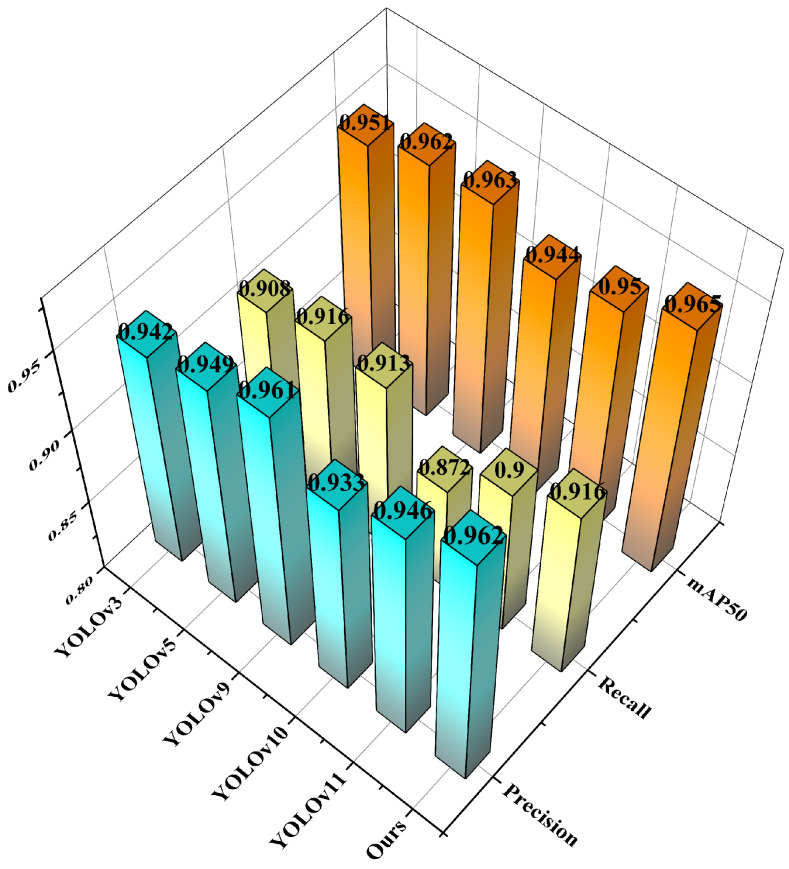
Comparison of training performance metrics for YOLO series models.

This study first compares our proposed YOLOv11-LSF network with other YOLO-series networks, including YOLOv3, YOLOv5, YOLOv9, YOLOv10, and the baseline YOLOv11, to evaluate its performance [22]. The experimental results of different models are presented in Table 4 and Figure 9.

In Table 4, we first compare the GFLOPs performance across the different YOLO versions. YOLOv3 exhibits a notably high computational complexity with 282.2 GFLOPs. In contrast, YOLOv5, YOLOv9, YOLOv10, and YOLOv11 demonstrate significantly improved efficiency in terms of GFLOPs. Specifically, YOLOv5 achieves 23.8 GFLOPs, YOLOv9 requires 26.7 GFLOPs, and YOLOv10 operates at 24.4 GFLOPs. Remarkably, YOLOv11 achieves the lowest computational complexity, with only 21.3 GFLOPs. Developers have continuously optimized the network architecture throughout the iterations of the YOLO-series models, reducing computational complexity to better adapt to real-time inference and low-power device requirements. Simultaneously, as shown in Figure 9, the bar chart plotted using the training metrics from this study exhibits relatively stable fluctuations. The training results of the selected YOLO-series models on the hybrid dataset demonstrate comparable performance in terms of precision and recall, with marginal differences in mAP across models. The YOLOv9 model achieved a mAP50 of 96.3%, making it the highest-accuracy model, excluding our improved version, while YOLOv10 had the lowest mAP50 at 94.4%, differing from YOLOv9 by 1.9%. Ultimately, we selected YOLOv11 as the baseline for this study due to its lightweight design and status as the latest version. As illustrated in Figure 9 and Table 4, YOLOv11’s training results underperform other selected network architectures in all metrics except model complexity, likely because competing architectures require greater computational resources to achieve higher accuracy. However, with the improvement method proposed in Section 3 of this study, YOLOv11 achieves state-of-the-art performance across all metrics while maintaining lightweight model complexity. It establishes an optimal balance between precision and computational cost, surpassing other network architectures comprehensively [23,24,25].

#### 4.4.2. Ablation Experiment

To visually evaluate the impact of different modules on model accuracy, we conducted a series of ablation studies based on YOLOv11 to demonstrate the efficacy of each component. First, we adopted YOLOv11s as a baseline, obtaining its detection results as the experimental reference. Next, we validated the effectiveness of individual modules by incrementally improving the baseline model with targeted modifications. We first improved the C2PSA module in the YOLOv11 backbone network while keeping the rest of the architecture unchanged. Experiments were conducted post-modification to ensure results were unaffected by external factors. This process was repeated iteratively to validate each improvement method independently. Finally, all proposed methods were integrated to construct the YOLOv11-LSF model, thereby proving its effectiveness [26].

To enhance the network’s ability to extract target object features and suppress background information, we introduced an attention mechanism into the C2PSA module of the backbone network, thereby improving recognition performance. To validate the superiority of the proposed LSKA attention module, we embedded different attention modules at the same location in the network architecture and conducted comparative experiments [27].

**Figure 10:**
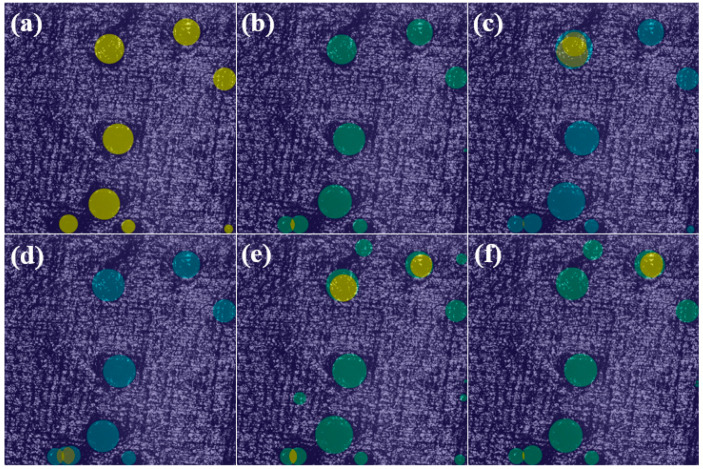
Comparison of heatmaps for multiple attention mechanisms: (**a**) YOLOv11s, (**b**) CA, (**c**) CBAM, (**d**) ECA, (**e**) GAM, (**f**) LSKA.

As shown in Table 5, embedding attention modules does not significantly increase parameter counts or computational costs. In comparison, the proposed C2PSA_LSKA module achieves the highest efficacy, improving mAP50 by 1.1% over the YOLOv11 baseline and by 1%, 0.8%, 15.5%, and 1.5% compared to CBAM, CA, GAM, and ECA modules, respectively. Furthermore, other attention mechanisms exhibit marginal or even degraded improvements in Precision, Recall, and mAP50-95 when compared to LSKA. For instance, integrating the CBAM module increases YOLOv11’s Precision, mAP50, and mAP50-95 by 0.6%, 0.1%, and 1.2%, respectively, but simultaneously reduces Recall by 0.07%. Consequently, the LSKA attention mechanism is selected for this study [28]. Meanwhile, Figure 10 presents the Heatmaps corresponding to various attention mechanisms evaluated in this study. The baseline YOLOv11 model without attention mechanisms fails to detect all damage precursors in the image, exhibiting missed detections (Figure 10a). Heatmaps from other attention mechanisms (Figure 10b–e) demonstrate less precise localization of damage precursors compared to the proposed LSKA (Figure 10f). This inaccuracy is most pronounced in the GAM-based results. In summary, the experimental results demonstrate that introducing the LSKA attention mechanism enhances the model’s focus on damage precursor regions while suppressing background interference. This is validated by the diffusion of network-regions interest in heatmaps generated with LSKA, which highlights its superior spatial attention allocation.

This study evaluates the effectiveness of four feature fusion networks: BiFPN, RepGFPN, ASF-YOLO, and Slim-neck. As shown in Table 6, compared to the original feature fusion network in YOLOv11, the proposed Slim-neck not only improves mAP50 and mAP50-95 by 1.3% and 2.9%, respectively, but also reduces model complexity by 0.2. The performance gain is attributed to the integration of GSConv and VoV-GSCSP structures in Slim-neck’s design. These components reduce the computational overhead of convolutional operations while enhancing the fusion efficiency of multi-scale feature maps. Consequently, Slim-neck achieves a lighter computational footprint without compromising detection accuracy [29]. The experimental results demonstrate the superiority of Slim-neck over other feature fusion networks. Specifically, Slim-neck achieves 1%, 2.1%, and 1.6% higher mAP50 compared to BiFPN, RepGFPN, and ASF-YOLO, respectively. The improvement in mAP50-95 is even more significant, with gains of 1.6%, 5.4%, and 3.1% over the same benchmarks. These results validate that Slim-neck enhances the model’s adaptability to multi-scale targets, which is critical for real-world detection tasks. Consequently, due to its outstanding performance, Slim-neck is selected as the neck network in our final model.

**Figure 11:**
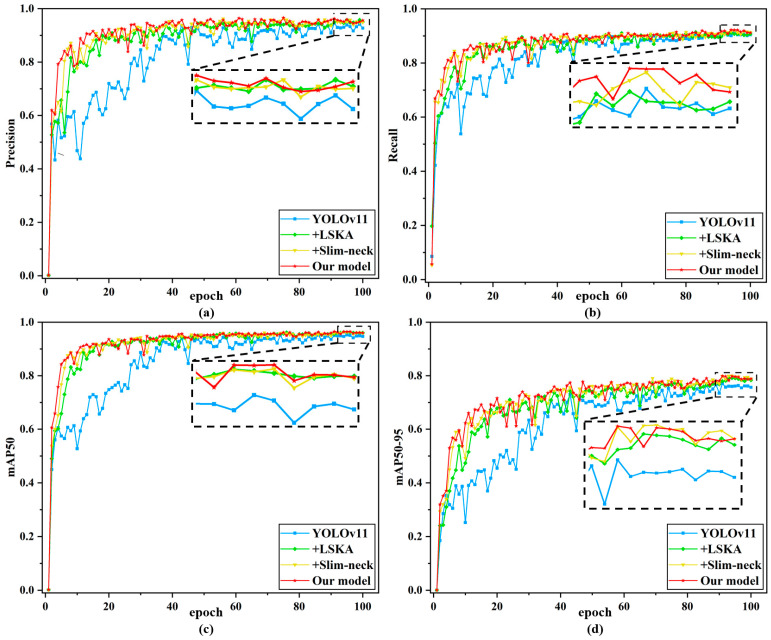
The ablation study results: (**a**) Precision curve; (**b**) Recall curve; (**c**) mAP50 curve; (**d**) mAP50-95 curve.

Based on the above experiments comparing different attention mechanisms and feature extraction networks, this study further conducts ablation experiments to validate the effectiveness of combining the LSKA attention mechanism with Slim-neck [30]. The results in Table 7 and Figure 11 confirm their synergistic contribution to model performance.

As clearly shown in Figure 11, the convergence speed of the original YOLOv11 network during training is suboptimal. For the four metrics evaluated, the baseline model requires at least 40 epochs to initiate convergence. In contrast, the proposed improvements significantly accelerate the stabilization of training curves for Precision, Recall, mAP50, and mAP50-95. This is evident in Figure 11a,c, where the curves begin to stabilize after only 20 epochs. Furthermore, the enhanced method consistently achieves the best performance across all training curves, demonstrating its superiority in balancing convergence efficiency and detection accuracy. As shown in Table 7, compared to YOLOv11, the introduction of the LSKA attention mechanism into the backbone network improves mAP50 by 1.1%, significantly enhancing model accuracy, but also increases GFLOPs by 0.7, raising model complexity. However, when Slim-neck is further integrated, GFLOPs are reduced to 21.7, while mAP50 continues to rise, reaching 96.5%, thereby optimizing computational resources without sacrificing accuracy. After all proposed improvements, YOLOv11-LSF achieves 1.6% higher Precision, 1.6% higher Recall, 1.5% higher mAP50, and 2.8% higher mAP50-95 compared to the baseline YOLOv11. As can be seen from the table, the improved approach proposed in this study enhances the detection accuracy for both categories of damage precursors, with a notable 2.8% increase in mAP50 for cracks, demonstrating significant improvement. The results demonstrate that the improved scheme enhances detection performance while preserving model lightweightness, optimizing YOLOv11 for surface damage precursor detection in additive manufacturing [31,32,33].

#### 4.4.3. Training Loss Comparison Curve

**Figure 12:**
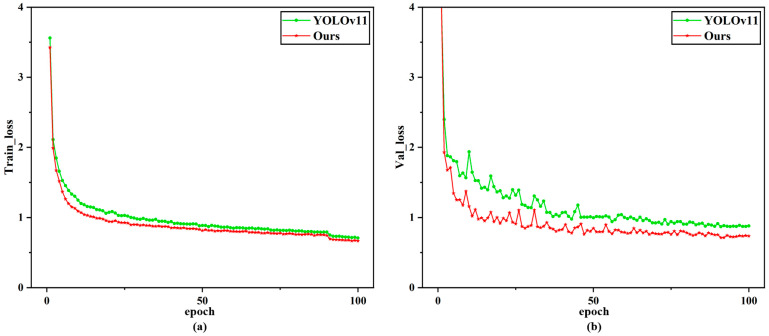
The cross-architecture training loss benchmarking curve: (**a**) training loss curve; (**b**) validation loss curve.

To further validate the effectiveness of the improved method on model convergence in this study, Figure 12 displays the loss convergence curves of the YOLOv11 model and our proposed model during training. By comparing the loss trajectories of both models, it is evident that the YOLOv11 model consistently exhibits higher loss values than the model proposed in this study throughout the training process. This discrepancy is most pronounced on the test set, where the loss value of YOLOv11-LSF reaches the final converged loss of the YOLOv11 model within approximately 10 epochs. Furthermore, it can be observed that the loss of the proposed model on the test set begins to stabilize around the 30th epoch, whereas the loss of YOLOv11 starts stabilizing after approximately the 45th epoch. Ultimately, the proposed model achieves training and test set loss values of 0.66538 and 0.73491, respectively, compared to the YOLOv11 model’s corresponding values of 0.70689 and 0.87955. These results conclusively demonstrate significant improvements in both stability and convergence for the enhanced model.

#### 4.4.4. Detection Results Benchmarking Across Model

**Figure 13:**
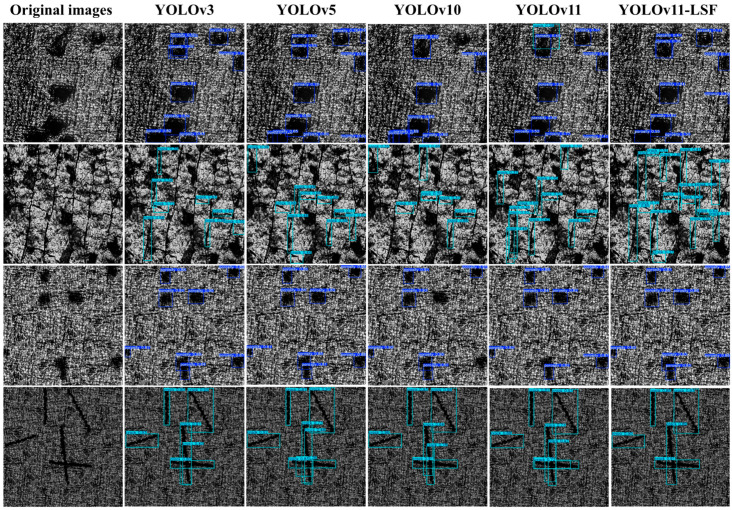
Test results of multiple models on simulated and captured data.

To more intuitively demonstrate the efficiency advantages of the improved method compared with other models, we conducted qualitative analysis through four representative damage precursor images selected from the test set. The first and second rows show the actual defect images acquired by the hardware equipment in this validation, while the third and fourth rows present the simulated results derived from the acquired images. Figure 13 presents the actual detection results of several advanced models on workpiece surface damage precursors. It should be noted that the six columns, respectively, represent: the original image, YOLOv3, YOLOv5, YOLOv10, YOLOv11, and the prediction results from our proposed mode.

As shown in the first row of Figure 13, the YOLOv11 model exhibits false detection phenomena when identifying porosity, misclassifying pores with lengths similar to cracks as cracks. In contrast, YOLOv11-LSF demonstrates immunity to such errors while achieving the highest confidence scores and most precise bounding box localization in porosity detection among all comparative models. By comparing the porosity detection results in the first and third rows, it can be observed that the models adopted in this study all demonstrate relatively accurate detection of porosity. Comparing the second and fourth rows reveals that on simulated scratch data, all other comparative models incorrectly segment single cracks into multiple detection fragments, whereas YOLOv11-LSF accurately identifies each complete crack instance without over-detection or classification errors. However, in the detection of actual captured scratch images, other comparative models exhibit severe missed detection issues for scratches, particularly evident in the results of YOLOv3, YOLOv5, and YOLOv10. Although YOLOv11-LSF still shows a performance gap compared to its test results on simulated data, the missed detection problem has been significantly mitigated relative to selected comparative models, with concurrently improved precision in bounding box localization. This study attributes the discrepancy between simulated and actual scratch detection to potential imperfections in crack background simulation within the current research framework, which will be further investigated in future work. The above analysis demonstrates that YOLOv11-LSF possesses stronger adaptive capability for detecting targets at different scales, which stems from our innovations in network architecture: the integration of LSKA in the backbone network and enhanced C2PSA mechanisms, enabling multi-scale target adaptation. These improvements collectively achieve precise simultaneous detection of both cracks and porosity, further validating our network’s robust multi-scale feature extraction and fusion capabilities [34].

## 5. Conclusions

This paper mainly focuses on enhancing detection accuracy and efficiency for surface damage precursors in additive manufacturing components by proposing YOLOv11-LSF, an efficient detection network. Experimental validations yield the following conclusions. First, regarding model training, we performed feature-level simulations of damage precursors in hardware-collected data, then implemented a hybrid training strategy combining captured and simulated datasets. As a result, we effectively resolved the challenge of insufficient training samples. Through quantitative experimental analysis, this study conducts comparative experiments between the improved model and YOLOv3, YOLOv5, YOLOv9, YOLOv10, and YOLOv11. The results demonstrate that the proposed YOLOv11-LSF model achieves the highest detection accuracy and optimal overall performance, with significant improvements in precision for various damage precursor categories. These findings further validate the feasibility and practicality of the YOLOv11-LSF algorithm in detecting surface damage precursors for additive manufacturing. Ablation experiments prove the effectiveness of each module. By improving the C2PSA module in the original network architecture, designing the cross-stage local network VoV-GSCSP using GSConv and a single-path aggregation strategy, and constructing the Slim-neck structure, the model achieves the best performance. Compared to YOLOv11, the proposed YOLOv11-LSF demonstrates improvements of 1.6% in precision, 1.6% in recall, 1.5% in mAP50, and 2.8% in mAP50-95, further confirming its superiority in detecting surface damage for additive manufacturing.

While the proposed model demonstrates strong adaptability to multi-scale targets, it still has certain limitations [35]. For instance, the model hits an impressive detection accuracy of 99% for porosity-related damage precursors, while its accuracy for cracks remains at 94%, indicating more work for improvement compared to porosity detection. The study attributes this discrepancy to two factors: cracks exhibit linear/dendritic/irregular shapes with micrometer-scale fissures, showing greater morphological diversity than porosity’s circular/elliptical pores. Such morphological diversity hinders the model from establishing unified feature representations. Additionally, cracks demonstrate greater randomness in length, width, and orientation, whereas porosity maintains at relatively stable sizes and shape distributions. These more complex scale variations substantially increase the model’s learning difficulty. Additionally, although the introduced attention mechanism enhances the model’s adaptability to multi-scale targets, it also increases model complexity. Therefore, we hope to design more lightweight attention mechanisms or methods in future work to strengthen the model’s capability to adapt to multi-scale targets.

## Figures and Tables

**Table 1:** Porosity and crack damage precursor images and their characteristic distributions.

Category	Captured Image	Damage Precursor Feature
porosity	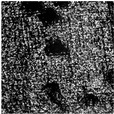	Circular spots, with black centers and relatively regular shapes
crack	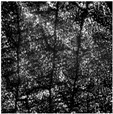	Light black, with distinct linear and branched fine lines intersecting each other.

**Table 2:** Experimental environment configuration.

Environment Configuration	Parameter
Operating system	Windows 11
CPU	Intel(R) Core (TM) i9-14900HX
GPU	NVIDIA GeForce RTX 4060(8 GB)
Development environment	Pycharm 2024.2.1
Language	Python 3.8.20
Operating platform	Pytorch2.4.1+cuda12.4

**Table 3:** Hyperparameter settings.

Hyperparameter	Parameter
Epochs	100
Batch size	16
Learning rate	0.01
Momentum	0.937
Weight decay	0.0005
Input image size	640

**Table 4:** The YOLO series network benchmarking results.

Model	GFLOPs	Precision	Recall	mAP50	mAP50-95
YOLOv3	282.2	94.2%	90.8%	95.1%	79.2%
YOLOv5	23.8	94.9%	91.6%	96.2%	77.6%
YOLOv9	26.7	96.1%	91.3%	96.3%	80.1%
YOLOv10	24.4	93.3%	87.2%	94.4%	79.8%
YOLOv11	21.3	94.6%	90%	95%	77.1%
Our model	21.7	96.2%	91.6%	96.5%	79.9%

**Table 5:** Comparison of the results of different attention mechanisms.

Model	GFLOPs	Precision	Recall	mAP50	mAP50-95
YOLOv11	21.3	94.6%	90%	95%	77.1%
+CBAM	21.5	95.2%	89.3%	95.1%	78.3%
+CA	21.3	95.9%	89%	95.3%	77.6%
+GAM	26.5	74.5%	84.2%	80.6%	53.6%
+ECA	21.3	94.8%	89.4%	94.6%	77.2%
+LSKA	22.0	94.9%	90.5%	96.1%	79.2%

**Table 6:** Results based on different feature fusion networks.

Model	GFLOPs	Precision	Recall	mAP50	mAP50-95
YOLOv11	21.3	94.6%	90%	95%	77.1%
+BiFPN	23.7	94.7%	89.5%	95.3%	78.4%
+RepGFPN	29.4	92.5%	90%	94.2%	74.6%
+ASF-YOLO	26.6	92.8%	90%	94.7%	76.9%
+Slim-neck	21.1	94.9%	91.6%	96.3%	80%

**Table 7:** Results of ablation experiment.

Method		AP		mAP50	mAP50-95	GFLOPs
LSKA	Slim-Neck	Porosity	Crack			
Baseline		98.8%	91.2%	95%	77.1%	21.3
**√**		99.3%	92.9%	96.1%	79.2%	22.0
	**√**	99.2%	93.3%	96.3%	80%	21.1
**√**	**√**	99%	94%	96.5%	79.9%	21.7

## Data Availability

The original contributions presented in this study are included in the article. Further inquiries can be directed to the corresponding author.
